# Superior vena cava thrombosis and dilated cardiomyopathy as initial presentations of Behcet’s disease

**DOI:** 10.1186/s12959-020-00225-y

**Published:** 2020-07-06

**Authors:** Ahmed M. Elzanaty, Mohammed T. Awad, Ashu Acharaya, Ebrahim Sabbagh, Eman Elsheikh, Moshrik AbdAlamir

**Affiliations:** 1 Internal Medicine Departement, University of Toledo, 3000 Arlington Avenue, Toledo, OH 43614 USA; 2 Cardiology Departement, University of Toledo, Toledo, Ohio USA; 3 Cardiology Departement, Tanta University Hospital, Tanta, Egypt

**Keywords:** SVC thrombosis, Dilated cardiomyopathy, Behcet’s disease

## Abstract

**Background:**

Bechet’s disease (BD) is a relatively rare disease that causes recurrent oral and genital ulcers in addition to a variety of systemic manifestations. Concomitant superior-vena-cava (SVC) thrombosis and cardiac involvement with dilated cardiomyopathy (DCM) as initial presentations for BD is considered rare.

**Case presentation:**

A 32-year-old-man presenting with intractable headaches and dyspnea. He was later diagnosed with SVC thrombosis and DCM. A diagnosis of BD was made after detailed history-taking.

**Conclusions:**

Cardiovascular manifisations can be the initial presentation of BD. We aim to highlight the importance of early clinical recognition of BD as a cause of DCM and SVC thrombosis.

## Introduction

BD is a rare disease, affecting 1 per 15,000 to 1 per 500,000 people in North America and European countries [1]. It is known to cause recurrent oral and genital ulcers alongside a wide variety of systemic presentation mostly related to vasculitis. Cardiac involvement, on the other hand, is rare in BD affecting from 7 to 31% of patients with the disease [2]. Cardiac involvement includes inflammation of all cardiac layers from the pericardium to myocardium, LV thrombus formation, endomyocardial fibrosis, and coronary arteritis [3–5].

## Case report

**Fig. 1:**
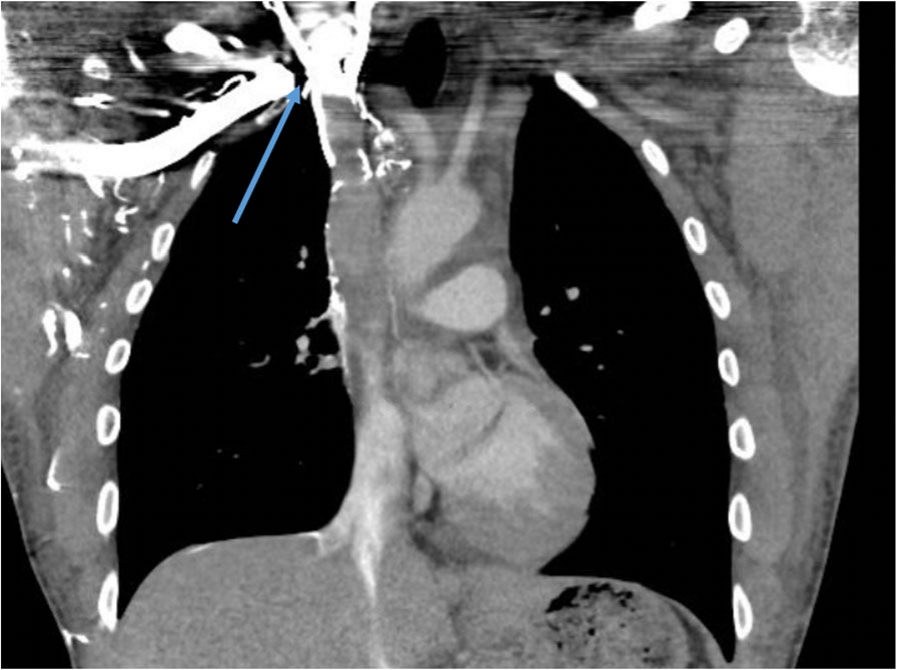
CT chest on admission showed SVC thrombus that extended to involve the brachiocephalic vein

A 32-year-old caucasian man with a past medical history of bronchial asthma presenting with generalized fatigue, orthopnea with intermittent fevers as well as recurrent sore throat for 9 months. Those symptoms triggered multiple emergency room visits and for which he received the diagnosis of recurrent viral upper respiratory tract infection (URTI). The patient started to develop intractable headaches with facial and chest wall swelling for 2 weeks prior to his admission to our hospital. Physical exam showed a positive Pemberton sign (facial plethora when raising the upper extremities). Lumbar puncture was performed which revealed a significantly elevated opening pressure of 36 mmH_2_0. With the suspicion of superior vena cava syndrome in mind, the patient subsequently underwent CT angiogram that confirmed the presence of SVC thrombosis that extended to involve the brachiocephalic vein (Fig. 1). Extensive investigations including autoimmune, vasculitis, as well as thrombophilia workup were done and they were within normal. The patient’s BNP, troponin I, and creatine kinase were also normal. The only lab abnormalities of significance were the raised ESR of 68 mm/, CRP of 7.3 mg/dl, anda reactive thrombocytosis with platelets of 506x10E9/L. The patient continued to have orthopnea despite initiation of heparin infusion and improvement of his SVC thrombosis symptoms for which a transthoracic echocardiogram was done which revealed global hypokinesia with ejection fraction (EF) of 20–25%.

**Fig. 2:**
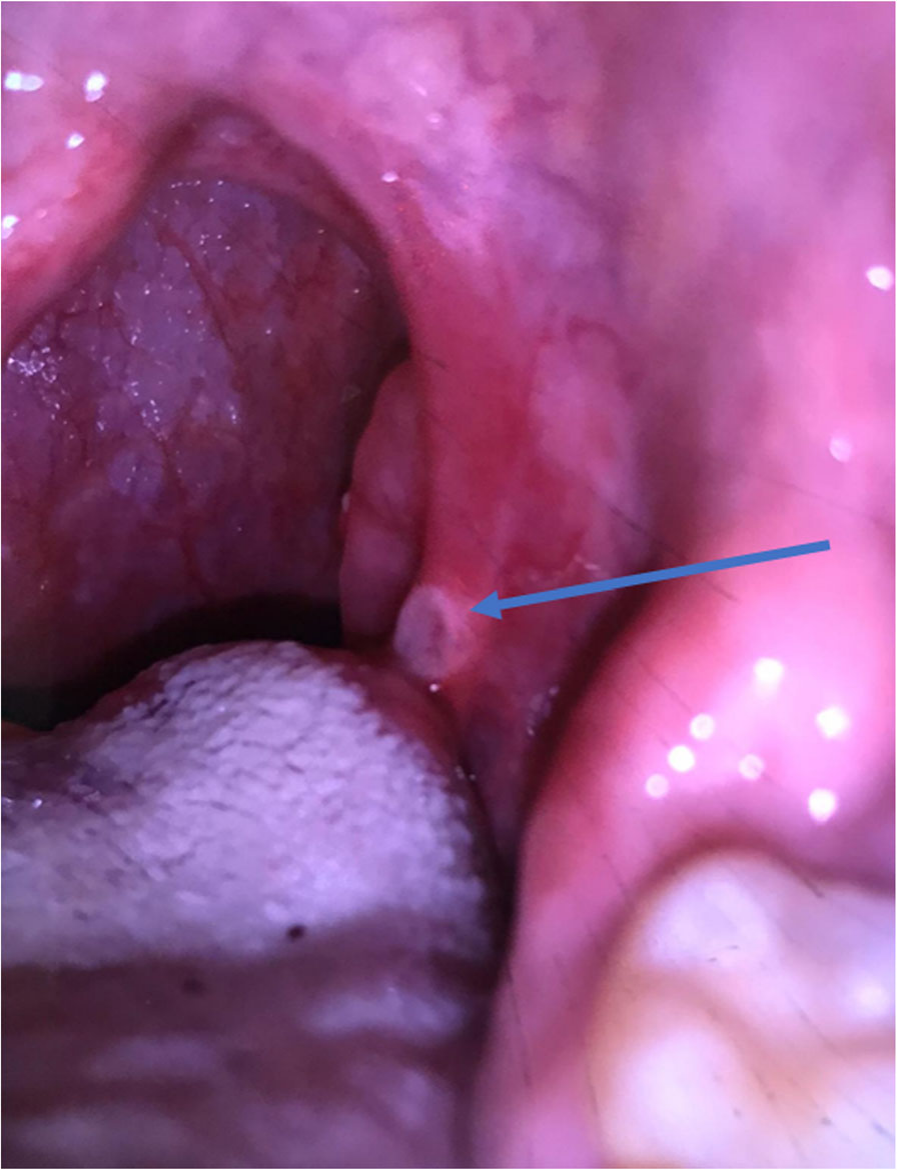
Self-captured photo for prior oral ulcer

**Fig. 3:**
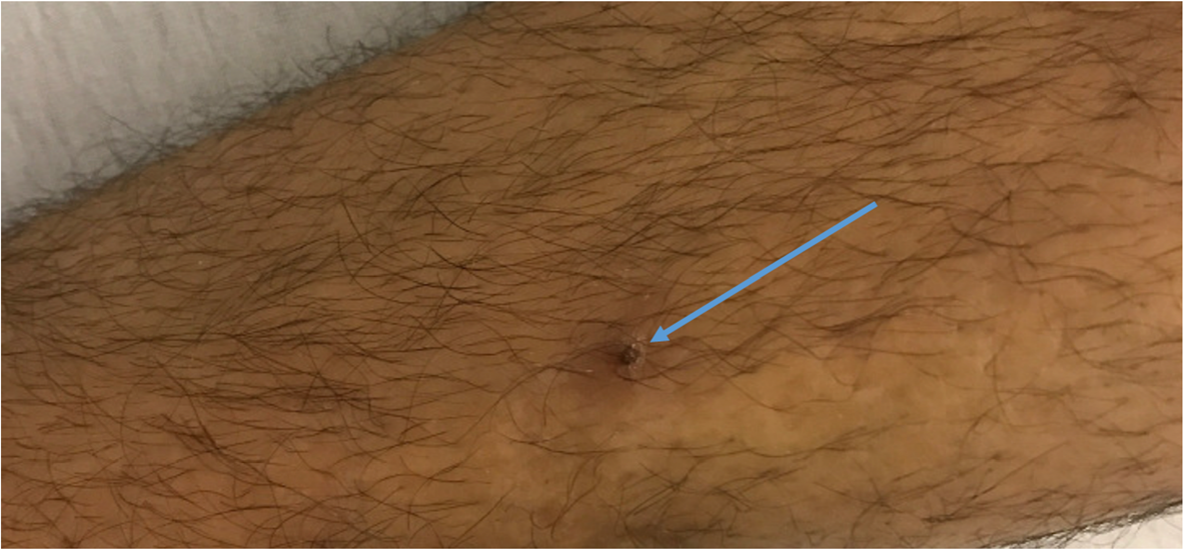
Photo of psudo-folliculitis lesion

Upon further detailed history taking, the patient reported having mouth ulcers that used to erupt whenever he had a sore throat (Fig. 2). He also reported unusual papules in his legs that matched the description of pseudofolliculitis (Fig. 3). Furthermore, he reported a family history of BD in one of his distant family members. Pathergy test was not formally done, but there were skin reactions reported after blood draws. A diagnosis of BD was made after fulfilling the diagnostic criteria of the International Study Group for BD with recurrent oral ulcers alongside skin lesions of pseudofolliculitis and positive pathergy test. The patient was then started on methylprednisone.

**Fig. 4:**
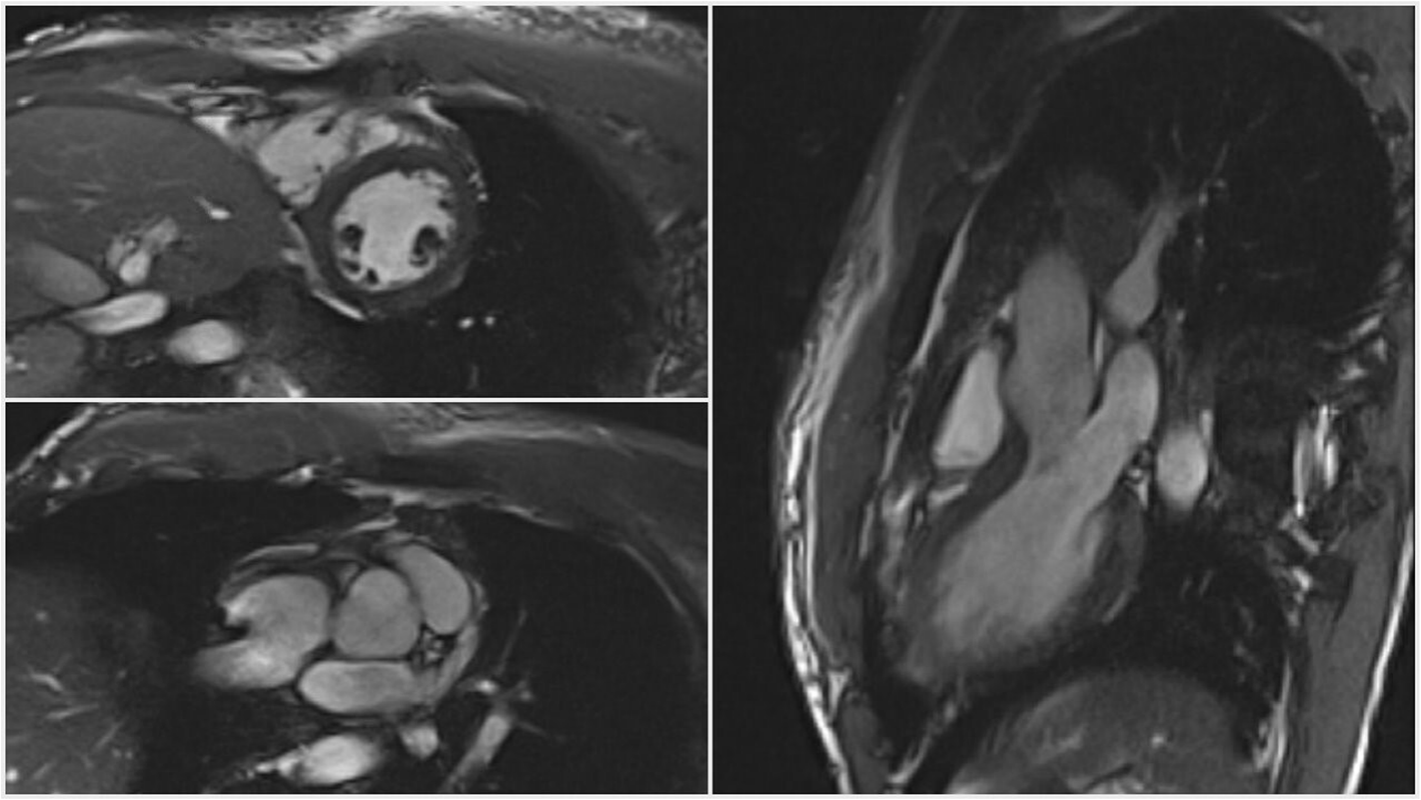
Multiple Sections through CMR revealing normal myocardium with no evidence of endomyocardial fibrosis or scarring

The patient was also started on sacubitril/valsartan, carvedilol, aspirin, and atorvastatin given his new heart failure with reduced ejection fraction. He later underwent myocardium perfusion imaging that showed low normal EF with no reversible ischemia making the diagnosis of coronary artery disease with ischemic cardiomyopathy less likely. Coronary angiography wasn’t pursued given low suspicion for coronary artery disease and on-going SVC thrombosis. Aspirin was also discontinued given the same reason. Three days after the initiation of steroids, the patient continued to report significant improvement in his symptoms, he eventually underwent cardiac MRI that revealed an improvement of his EF to 52% with no evidence of myocardial scarring or fibrosis (Fig. 4). The patient was discharged on oral prednisone, lisinopril, metoprolol succinate, and warfarin. The patient was in good condition upon discharge with follow-up with rheumatology, vascular surgery, and cardiology for his BD, SVC thrombosis, and heart failure with recovered ejection fraction.

On outpatient follow up, the patient remained to do well. His steroids were gradually tapered and he was started on colchicine after which he continued to be symptom-free.

## Case discussion

BD is a chronic relapsing autoimmune disorder of unknown etiology that is rare especially outside of the Silkroad area [6]. It is mostly diagnosed clinically, and that is why a thorough history and physical exam are needed to uncover it. A diagnosis is made when a patient is found to have recurrent oral ulcers alongside two of the following; recurrent genital aphthae, eye lesions like anterior or posterior uveitis, skin lesions like pseudofolliculitis, and a positive pathergy test [7].

BD’s main pathophysiology is through vascular involvement [8], in the form of vasculitis, thromboembolic complication, as well as pseudoaneurysm [9]. Venous involvement is believed to be secondary to endothelial inflammation leading to eventual thrombosis [10]. Vascular involvement can be effectively managed with immunosuppression with anticoagulation being preserved if thrombotic events occurred [11]. In our case, the main symptom that prompted the patient to seek medical attention was the protracted headache, which was later attributed to increased intracranial pressure secondary to SVC thrombosis. SVC involvement in BD is well known, Koc et al. in his review of vascular involvement in BD, report it as the 3rd most common venous site involvement [12]. The patient continued to have orthopnea and shortness of breath despite SVC thrombosis treatment for which a 2D echo was done that lead to the incidental discovery of DCM.

Cardiac involvement in BD is rare but reported in the medical literature [13, 14]. The cardiac presentation includes inflammation of one or all of cardiac layers, endomyocardial fibrosis, coronary arteritis with subsequent coronary artery disease, intracardiac thrombus, conduction system disturbances, and valvular disease [15]. Patients who have underlying cardiac complications have a poor prognosis with mortality reaching 20% [15]. However, to our knowledge the association of non-ischemic DCM with BD has been rarely reported with less than a handful of reported cases being found in our search [16–18]. In our case, the patient was started early on steroids with a rapid and remarkable recovery of the symptoms as well the myocardium function over the course of 3 days, with symptoms as well as ejection fraction recovering from 25% on 2D echo to 52% on Cardiac MRI. Our case is different from prior case reports as it might point to the fact that early steroid use can rapidly convert heart failure with reduced ejection fraction to heart failure with recovered EF.

In our case, if an early emphasis was made on history taking as well as a detailed physical exam to uncover the recurrent oral ulcers alongside the skin lesions instead of initially fixating on the diagnosis of viralURTI would have saved the patient recurrent ER visits.

## Conclusion

Recognizing the rare, but possibly grave, cardiac manifestation of BD including dilated non-ischemic cardiomyopathy is essential as it might aid in making the diagnosis and avoid the burden of over-testing.

## Learning objectives

1. Recognize cardiovascular manifestation of BD including DCM and SVC thrombosis.
2. Emphasis the role of history taking in cases of unexplained cardiomyopathy to avoid the burden of over-testing.

## Data Availability

Not applicable.

## Abbreviations

DCM: Dilated cardiomyopathy
BD: Behcet’s disease
SVC: Superior vena cava
